# Efficacy and Safety of Ertugliflozin Compared to Placebo in Patients With Type 2 Diabetes: An Updated Systematic Review and Meta-Analysis

**DOI:** 10.1155/2024/5553327

**Published:** 2024-09-24

**Authors:** A. B. M. Kamrul-Hasan, Muhammad Shah Alam, Samir Kumar Talukder, Mohammad Abdul Hannan, Deep Dutta, Lakshmi Nagendra, Shahjada Selim

**Affiliations:** ^1^ Department of Endocrinology Mymensingh Medical College, Mymensingh, Bangladesh; ^2^ Department of Medicine Army Medical College Cumilla, Cumilla, Bangladesh; ^3^ Department of Endocrinology Rangpur Medical College, Rangpur, Bangladesh; ^4^ Department of Endocrinology North East Medical College and Hospital, Sylhet, Bangladesh; ^5^ Department of Endocrinology CEDAR Superspeciality Healthcare, Dwarka, New Delhi, India; ^6^ Department of Endocrinology JSS Medical College JSS Academy of Higher Education and Research, Mysore, India; ^7^ Department of Endocrinology Bangabandhu Sheikh Mujib Medical University, Dhaka, Bangladesh

**Keywords:** ertugliflozin, glycemic efficacy, hypoglycemia, meta-analysis, Type 2 diabetes

## Abstract

**Background:** No comprehensive meta-analysis has evaluated the efficacy and safety of ertugliflozin compared to a placebo in patients with Type 2 diabetes (T2D) until now. This meta-analysis fills this gap in knowledge.

**Methods:** A systematic search was carried out in electronic databases to identify randomized controlled trials (RCTs) that included patients with T2D receiving ertugliflozin in the treatment group and placebo in the control group. The change in HbA1c from the baseline values was the primary outcome, whereas changes in plasma glucose and other metabolic parameters and adverse events (AEs), including hypoglycemia, were the secondary outcomes.

**Results:** Seven RCTs involving 7283 subjects met the inclusion criteria. Ertugliflozin outperformed placebo in reducing HbA1c in both 5 mg (MD −0.62%, 95% CI [−0.80, −0.44], *p* < 0.00001, *I*^2^ = 91%) and 15 mg (MD −0.69%, 95% CI [−0.91, −0.47], *p* < 0.00001, *I*^2^ = 93%) doses. A higher proportion of patients achieved HbA1c < 7.0% with ertugliflozin than with placebo. Ertugliflozin was also superior to placebo in lowering fasting plasma glucose (FPG), body weight, and systolic and diastolic blood pressure (BP). Ertugliflozin and placebo had comparable AE profiles, including urinary tract infection (UTI) and hypoglycemia, except for the greater risk of genital mycotic infections (GMIs) with ertugliflozin. Ertugliflozin 5 and 15 mg have equivalent efficacy and safety profiles except for greater weight reduction with ertugliflozin 15 mg.

**Conclusion:** Ertugliflozin has a good glycemic efficacy and a reassuring safety profile in managing T2D.

**Trial Registration:** Registration number: CRD42023456450.

## 1. Introduction

Sodium-glucose cotransporter 2 (SGLT2) inhibitors (SGLT2is) inhibit glucose reabsorption at the renal proximal convoluted tubules, causing glucose loss through urine and thus lowering plasma glucose [1]. These drugs have shown solid evidence of extraglycemic merits of reducing cardiovascular (CV) mortality and heart failure-related hospitalization in patients with Type 2 diabetes (T2D) having established CV disease (CVD) or at high atherosclerotic CVD (ASCVD) risk [2]. SGLT2is also slow the progression of chronic kidney disease (CKD) in patients with T2D at high CV risks [3]. Furthermore, they have the potential for weight loss and blood pressure (BP) reductions [1]. For such proven benefits, SGLT2is are recommended as part of the glucose-lowering regimen independent of glycemic control and metformin use for T2D patients with established or high risk of ASCVD, heart failure, or CKD [4]. Commonly reported adverse events (AEs) associated with SGLT2i use include pollakiuria, genital mycotic infections (GMIs), and urinary tract infections (UTIs) [5]. SGLT2i use is growing worldwide, particularly in populations with higher CV risk [6, 7]. To date, there are five SGLT2is approved by the Food and Drug Administration (FDA) for use in adults with T2D, namely, canagliflozin, dapagliflozin, empagliflozin, ertugliflozin, and bexagliflozin. Ertugliflozin is relatively newer among these, and the FDA approved it in December 2017 [8].

Many clinical trials and observational studies have reported the efficiency and safety of ertugliflozin in T2D, and there are marked heterogeneities in the outcome data. Although some meta-analyses have been conducted to summarize the efficacy and safety data of ertugliflozin, the meta-analyses incorporated original studies along with their follow-up reports together in the same analysis; they also did not perform separate analyses of placebo and active comparators to compare with ertugliflozin [9–11]. With this background, this meta-analysis was conducted to holistically assess the effectiveness and safety of ertugliflozin compared to placebo in managing T2D, incorporating all the available randomized controlled trials (RCTs).

## 2. Materials and Methods

This meta-analysis strictly complied with the guidelines described in the Cochrane Handbook for Systematic Reviews of Interventions and the Preferred Reporting Items for Systematic Reviews and Meta-Analyses (PRISMA) checklists [12]. The protocol summary was officially recorded in PROSPERO. This meta-analysis required no additional ethical approval since such approval had already been obtained for the individual included studies.

A thorough systematic search was conducted through multiple databases and registers, including MEDLINE (via PubMed), Scopus, Google Scholar, Cochrane Central Register, ClinicalTrials.gov, and the International Clinical Trials Registry Platform (ICTRP). The search covered these sources' inception until 10 October 2023. The search strategy utilized a Boolean approach with the terms (ertugliflozin) AND (type 2 diabetes); the search terms were applied to the titles and abstracts. A careful and thorough search was conducted to find published or unpublished clinical trials in English; this search included examining references within the RCTs included in this meta-analysis and relevant journals.

The selection of RCTs for this meta-analysis was based on the PICOS criteria. The patient population (P) consisted of individuals with T2D; the intervention (I) was the administration of ertugliflozin for managing T2D; the control (C) included individuals receiving placebo; the outcomes (O) included glycated hemoglobin (HbA1c); and the study type (S) included the RCTs. This meta-analysis included RCTs conducted among adults (aged ≥ 18 years) with T2D and a minimum 12-week duration. The trials must have at least two arms/groups of intervention, with one of ertugliflozin as monotherapy or as part of a standard diabetes treatment regimen and the other of a placebo, alone or in combination. Exclusion criteria were the clinical trials among animal or healthy humans, nonrandomized trials, retrospective studies, case reports, letters to editors, articles not having data with outcomes of interest, and RCTs < 12 weeks in duration.

The primary outcome of this meta-analysis was the change from the baseline in HbA1c at the end of the trial. The secondary outcomes were the proportion of the study subjects achieving HbA1c < 7.0% at the end of the trial, the changes from the baseline in fasting plasma glucose (FPG), body weight, systolic BP, diastolic BP, low-density lipoprotein cholesterol (LDL-C), high-density lipoprotein cholesterol (HDL-C), and AEs, including GMI in women and men, and hypoglycemia. The outcome analysis was further stratified according to the dose of ertugliflozin (5 mg or 15 mg).

Four authors independently extracted data using standardized forms, with details provided elsewhere [13]. The handling of missing data has also been elaborated in the same source [13]. Four authors independently performed the risk of bias (RoB) assessment using the RoB tool in Review Manager (RevMan), version 7.2.0 (The Cochrane Collaboration, 2024. Available at revman.cochrane.org). The same source also outlined the specific biases [13].

For analysis, we expressed HbA1c as %, plasma glucose as mmol/L, body weight as kg, BP as mmHg, and cholesterol as mg/dL. The results obtained from studies implemented with varying units were converted for optimal consistency by applying appropriate conversion factors. The results of the outcomes were expressed as mean differences (MDs) for continuous variables and as odds ratios (ORs) or risk ratios (RRs) for dichotomous variables with 95% confidence intervals (CIs). The RevMan, version 7.2.0 (The Cochrane Collaboration, 2024. Available at revman.cochrane.org), was used to compare MD and OR (or RR) for the primary and secondary outcomes between the ertugliflozin and placebo groups. Random-effect models were chosen for the review to account for the expected heterogeneity arising from differences in population characteristics and research durations. Forest plots, created using RevMan version 7.2.0 (The Cochrane Collaboration, 2024. Available at revman.cochrane.org), portrayed outcomes, with the left side favoring ertugliflozin and the right side favoring the placebo. A significance level of *p* < 0.05 was used. The results included forest plots incorporating data from at least two RCTs.

Initially, the heterogeneity was evaluated by analyzing forest plots. After that, a Chi^2^ test was conducted with N-1 degrees of freedom and a significance level of 0.05 to ascertain the statistical significance. Additionally, the *I*^2^ test was utilized in further analysis [14]. The details of interpreting *I*^2^ values have already been elaborated elsewhere [13].

The quality of the evidence of the important outcomes of this meta-analysis was evaluated using the Grading of Recommendations Assessment, Development and Evaluation (GRADE) approach [15]. The detailed process of formulating the summary of finding (SOF) table and labeling the quality of evidence as “high,” “moderate,” “low,” or “very low” has been previously described [13].

## 3. Results

### 3.1. Search Results

**Figure 1:**
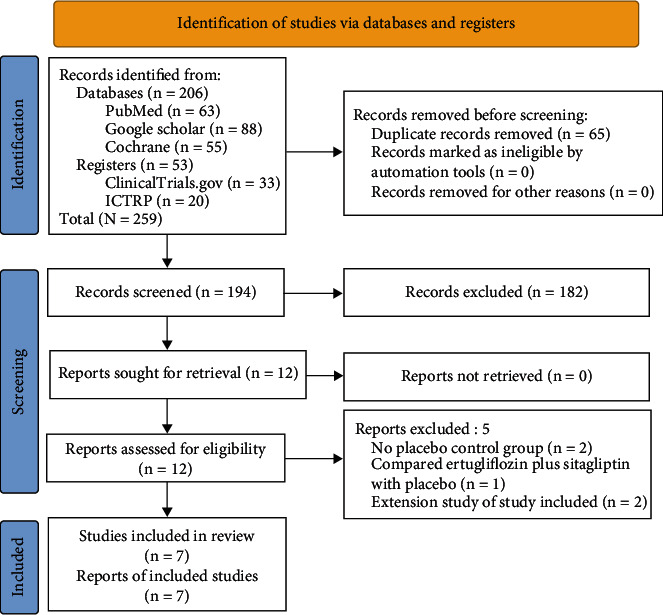
Flowchart on study retrieval and inclusion in the meta-analysis.

Figure 1 depicts the study selection process. After the initial search, 259 articles were found; after screening the titles and abstracts, followed by full-text checking, the search was reduced to 12 studies evaluated in detail for inclusion in this meta-analysis. Finally, seven RCTs, which satisfied all criteria, were included and analyzed in this meta-analysis [16–22]. Five initially selected studies were excluded because they either had no placebo-control group [23, 24], compared ertugliflozin plus sitagliptin with placebo [25], or were reports of the extension studies of the trials included in this meta-analysis [26, 27]. In multiphasic studies, we incorporated only the placebo-controlled phase in this meta-analysis [18–20, 22].

### 3.2. Study Characteristics

Of the seven RCTs in this meta-analysis, subgroup analyses were performed based on the dose of ertugliflozin used in the intervention arm, 5 mg or 15 mg. All but one study by Amin et al. had both ertugliflozin 5 mg and 15 mg in the intervention arms [16]. In the forest plot, the outcomes of ertugliflozin 5 mg versus placebo and ertugliflozin 15 mg versus placebo were analyzed separately; suffix “a” and “b” after the study ID (e.g., Cannon 2020a and Cannon 2020b [17]) indicated the subgroups of the study with ertugliflozin 5 mg and ertugliflozin 15 mg in the intervention groups, respectively. The summary of the included and excluded studies is given in Tables 1 and 2, respectively.

### 3.3. RoB in the Included Studies

**Figure 2:**
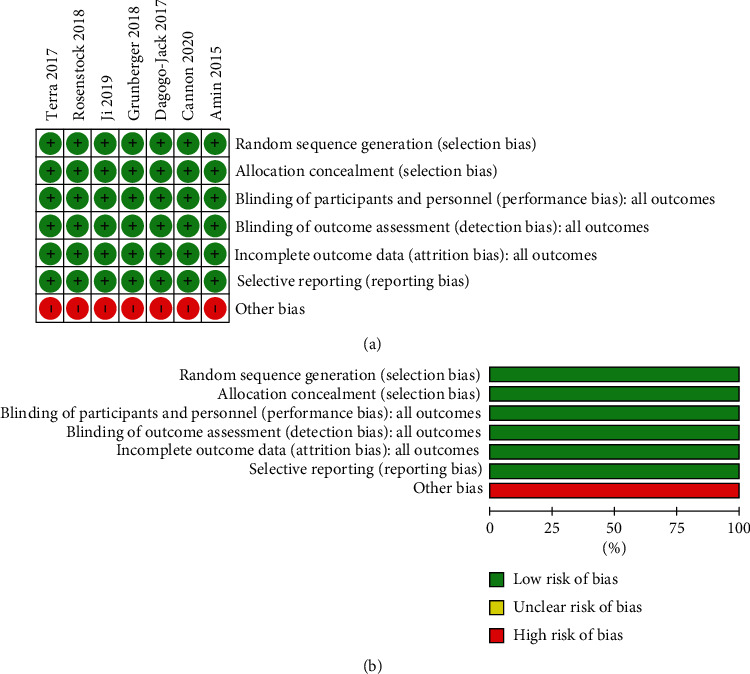
(a) Risk of bias summary: review authors' judgments about each risk of bias item for each included study; (b) risk of bias graph: review authors' judgments about each risk of bias item presented as percentages across all included studies.

Figure 2 depicts the RoB across the seven studies included in the meta-analysis. All seven studies (100%) exhibited a low risk of selection bias, performance bias, detection bias, attrition bias, and reporting bias; however, all (100%) had a high risk of other biases. The detailed process of bias risk assessment is available as a supplementary file (Table S1).

### 3.4. Grading of the Results

The grades of the certainty of evidence of the results are given in the SOF table (Table 3).

### 3.5. Effect of Ertugliflozin on the Glycemic Parameters

**Figure 3:**
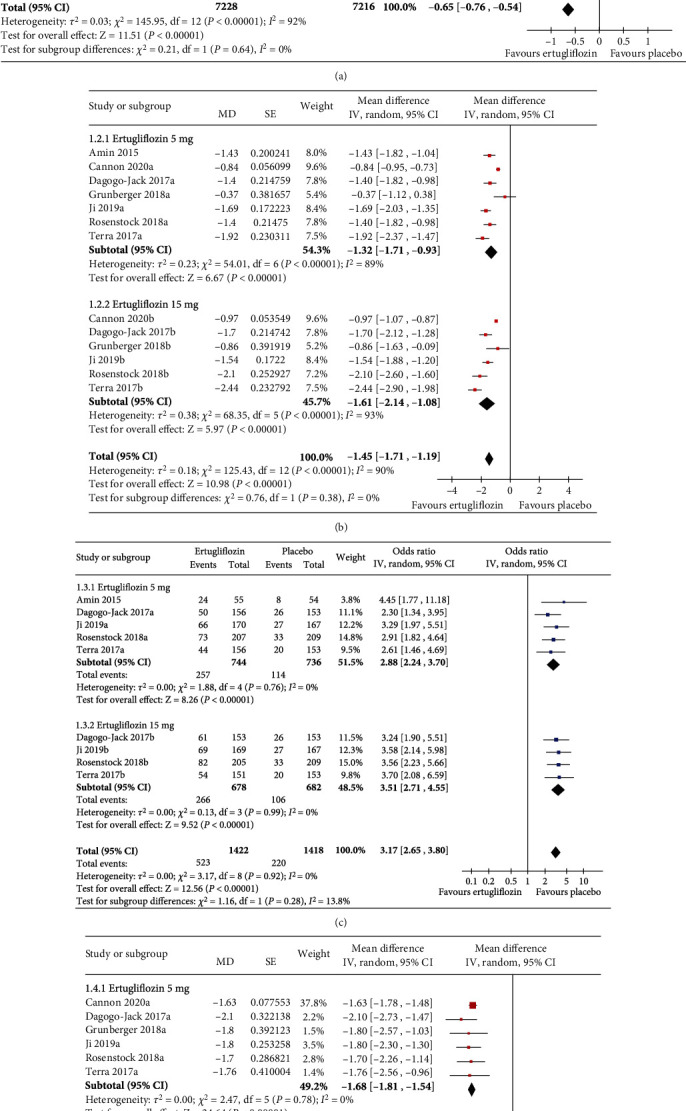
Forest plot highlighting the impact of ertugliflozin on the (a) change in HbA1c, (b) change in FPG, (c) the proportion of the study subjects who achieved HbA1c < 7.0%, and (d) change in body weight from baseline as compared to placebo. CI = confidence interval; df = degrees of freedom; FPG = fasting plasma glucose; HbA1c = glycated hemoglobin; IV = inverse variance; MD = mean difference; SD = standard deviation; SE = standard error.

Seven studies (*n* = 7283) with ertugliflozin 5 mg and six (*n* = 7161) with ertugliflozin 15 mg reported changes in HbA1c. Compared to the placebo, greater reductions in HbA1c were achieved with both ertugliflozin 5 mg (MD −0.62%, 95% CI [−0.80, −0.44], *p* < 0.00001, *I*^2^ = 91%) and ertugliflozin 15 mg (MD −0.69%, 95% CI [−0.91, −0.47], *p* < 0.00001, *I*^2^ = 93%) (Figure 3(a)). Ertugliflozin also outperformed placebo in reductions of FPG at both doses: for 5 mg (MD −1.32 mmol/L, 95% CI [−1.71, −0.93], *p* < 0.00001, *I*^2^ = 89%) and 15 mg (MD −1.61 mmol/L, 95% CI [−2.14, −1.08], *p* < 0.00001, *I*^2^ = 93%) (Figure 3(b)). The percentages of subjects achieving HbA1c < 7% were also higher in the empagliflozin arms: for 5 mg (OR 2.88, 95% CI [2.44, 3.70], *p* < 0.00001, *I*^2^ = 0%) and 15 mg (OR 3.51, 95% CI [2.71, 4.55], *p* < 0.00001, 0%) doses (Figure 3(c)).

### 3.6. Effect of Ertugliflozin on Other Metabolic Parameters

**Figure 4:**
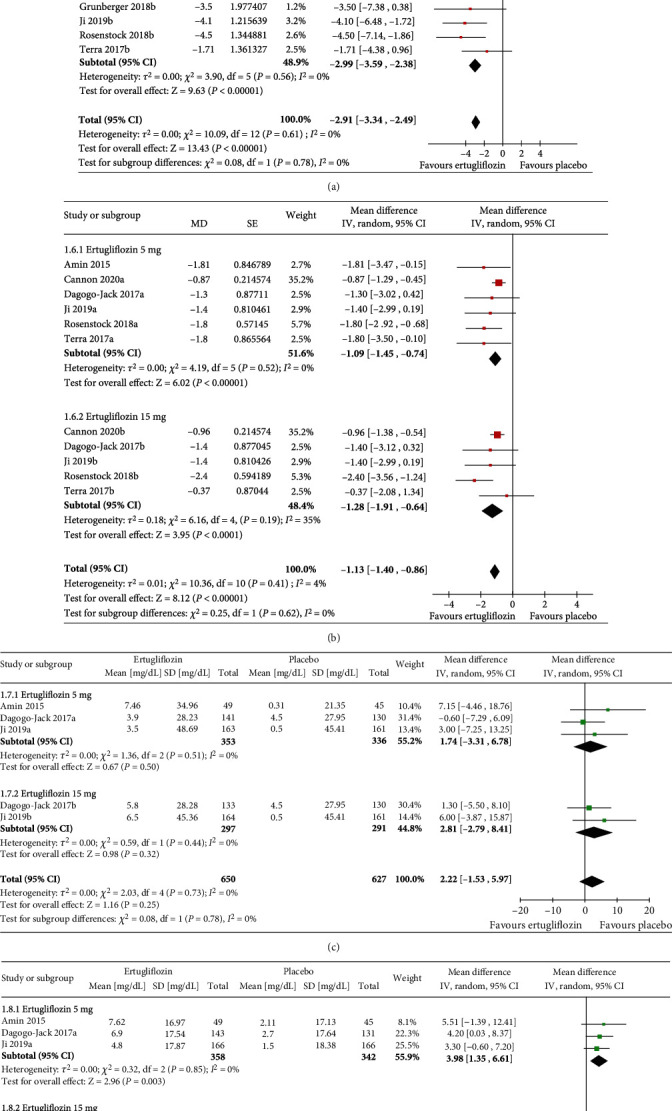
Forest plot highlighting the impact of ertugliflozin on changes in the (a) SBP, (b) DBP, (c) LDL-C, and (d) HDL-C from baseline compared to placebo.

Greater reductions in body weight were achieved with both ertugliflozin 5 mg (MD −1.68 kg, 95% CI [−1.81, −1.54], *p* < 0.00001, *I*^2^ = 0%) and ertugliflozin 15 mg (MD −1.90 kg, 95% CI [−2.04, −1.77], *p* < 0.00001, *I*^2^ = 0%) (Figure 3(d)) than placebo. Reductions in SBP were higher with both ertugliflozin 5 mg (MD −2.86 mmHg, 95% CI [−3.48, −2.24], *p* < 0.00001, *I*^2^ = 1%) and ertugliflozin 15 mg (MD −2.99 mmHg, 95% CI [−3.59, −2.38], *p* < 0.00001, *I*^2^ = 0%) (Figure 4(a)). Similarly, ertugliflozin was superior to placebo in reducing DBP at both doses (for ertugliflozin 5 mg, MD −1.09 mmHg, 95% CI [−1.45, −0.74], *p* < 0.00001, *I*^2^ = 0%), and for ertugliflozin 15 mg, MD −1.28 mmHg, 95% CI [−1.91, −0.64], *p* < 0.00001, *I*^2^ = 4%) (Figure 4(b)). The overall effect of ertugliflozin on LDL-C was statistically not significant; for 5 mg (MD 1.74 mg/dL, 95% CI [−3.31, 6.78], *p* = 0.50, *I*^2^ = 0%) and 15 mg (MD 2.81 mg/dL, 95% CI [−2.79, 8.41], *p* = 0.32, *I*^2^ = 0%) (Figure 4(c)). Elevations in HDL-C were higher with both ertugliflozin 5 mg (MD 3.98 mg/dL, 95% CI [1.35, 6.61], *p* = 0.003, *I*^2^ = 0%) and with ertugliflozin 15 (MD 3.28 mg/dL, 95% CI [0.31, 6.24], *p* = 0.03, *I*^2^ = 0%) than placebo (Figure 4(d)).

### 3.7. AEs

**Figure 5:**
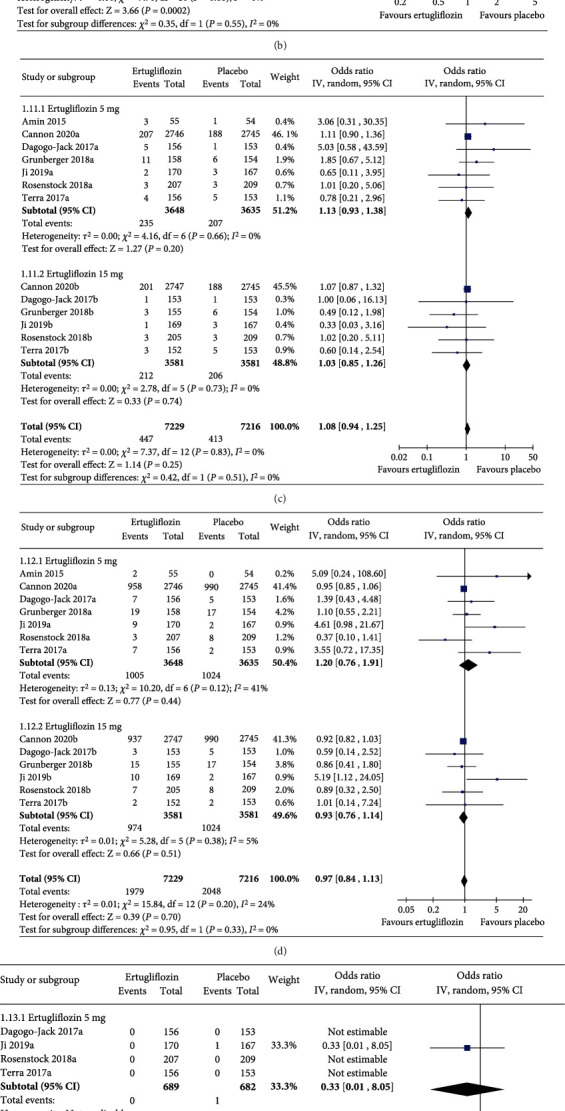
Forest plot highlighting the events of (a) any AEs, (b) AEs related to the study drug, (c) AEs leading to discontinuation of the study drug, (d) serious AEs, (e) serious AEs related to the study drug, and (f) AEs leading to death with ertugliflozin compared to placebo.

Compared to placebo, ertugliflozin 5 mg and 15 mg did not increase the risks of any AEs (Figure 5(a)), AEs leading to discontinuation of the study drug (Figure 5(c)), serious AEs (Figure 5(d)), serious AEs related to the study drug (Figure 5(e)), and AEs leading to death (Figure 5(f)). The risk of AEs related to the study drug was higher with ertugliflozin 5 mg than with placebo (OR 1.58, 95% CI [1.18, 2.10], *p* = 0.002, *I*^2^ = 0%); however, such risk was similar with ertugliflozin 15 mg and placebo (Figure 5(b)).

#### 3.7.1. GMIs

**Figure 6:**
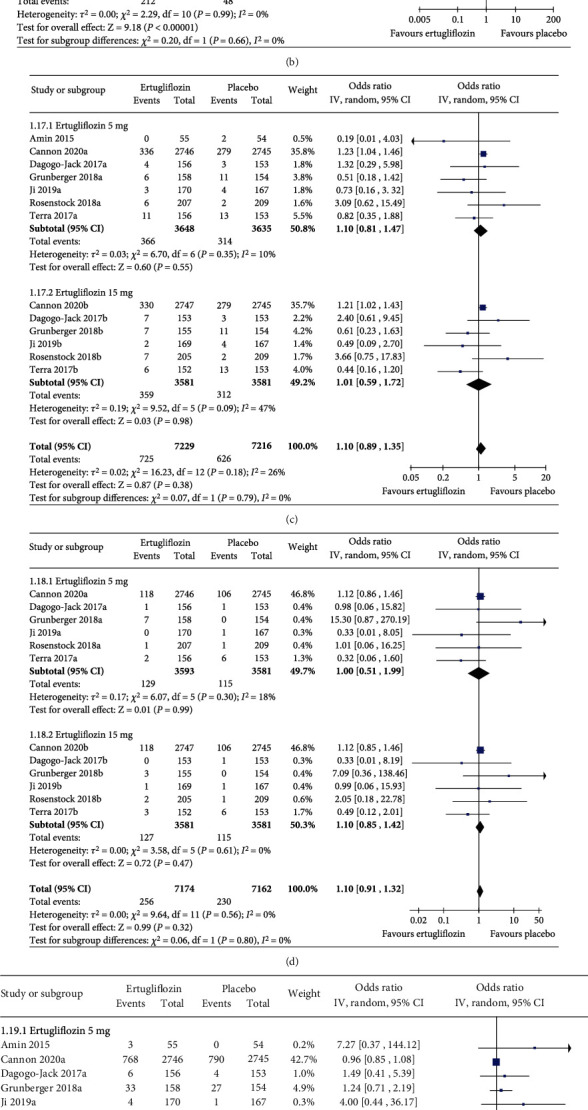
Forest plot highlighting the events of (a) GMI (women), (b) GMI (men), (c) UTI, (d) hypovolemia, (e) symptomatic hypoglycemia, and (f) documented hypoglycemia with ertugliflozin compared to placebo.

The risks of GMI were higher with both ertugliflozin 5 mg and 15 mg than placebo in women (for 5 mg, OR 2.95, 95% CI [1.88, 4.62], *p* < 0.00001, *I*^2^ = 0%, and for 15 mg, OR 3.79, 95% CI [2.45, 5.87], *p* < 0.00001, *I*^2^ = 0%) (Figure 6(a)) and men (for 5 mg, OR 3.99, 95% CI [2.57, 6.19], *p* < 0.00001, *I*^2^ = 0%, and for 15 mg, OR 4.59, 95% CI [2.96, 7.11], *p* < 0.00001, *I*^2^ = 0%) (Figure 6(b)).

#### 3.7.2. UTIs

Compared to placebo, ertugliflozin did not increase the risks of UTI (for 5 mg: OR 1.10, 95% CI [0.81, 1.47], *p* = 0.55, *I*^2^ = 10%; and for 15 mg: OR 1.01, 95% CI [0.59, 1.72], *p* = 0.98, *I*^2^ = 47%) (Figure 6(c)).

#### 3.7.3. Hypovolemia

The risk of hypovolemia was also not higher with both ertugliflozin 5 mg (OR 1.00, 95% CI [0.51, 1.99], *p* = 0.99, *I*^2^ = 18%) and 15 mg (OR 1.10, 95% CI [0.85, 1.42], *p* = 0.47, *I*^2^ = 0%) than with placebo (Figure 6(d)).

#### 3.7.4. Symptomatic and Documented Hypoglycemia

Both ertugliflozin 5 mg and 15 mg had similar risks of symptomatic hypoglycemic events to placebo (for 5 mg: OR 0.99, 95% CI [0.88, 1.10], *p* = 0.81, *I*^2^ = 0%, and for 15 mg: OR 1.06, 95% CI [0.69, 1.62], *p* = 0.80, *I*^2^ = 36%) (Figure 6(e)). The risk of documented symptomatic hypoglycemic events was similar with ertugliflozin 5 mg and placebo (OR 1.91, 95% CI [0.98, 3.73], *p* = 0.06, *I*^2^ = 0%; such risk was higher with ertugliflozin 15 mg (OR 2.24, 95% CI [1.16, 4.31], *p* = 0.02, *I*^2^ = 0%) than placebo (Figure 6(f)).

### 3.8. Comparison of Ertugliflozin 5 mg and 15 mg for Efficacy and Safety Outcomes

Ertugliflozin 5 mg and 15 mg had similar efficacy compared to placebo in reducing HbA1c, FPG, percentage of subjects achieving HbA1c < 7%, SBP, and DBP; higher body weight reduction was accomplished with ertugliflozin 15 mg (0.22 kg higher, *p* = 0.020) than ertugliflozin 5 mg. Both doses had similar safety profiles compared to placebo.

## 4. Discussion

This meta-analysis incorporates the results of clinical trials of ertugliflozin in T2D published to date. It reports the glycemic efficacy, AEs, and hypoglycemic potential of ertugliflozin compared to a placebo used as an add-on therapy to lifestyle modifications (diet and exercise) with/without other glucose-lowering drugs. Ertugliflozin is more efficacious in HbA1c reduction than placebo and has a safety profile comparable to placebo except for GMI.

Ertugliflozin is a highly selective inhibitor of SGLT2 with > 2000-fold selectivity for SGLT2 over SGLT1. Its oral bioavailability is ~100%, higher than that of canagliflozin (65%), dapagliflozin (78%), and empagliflozin (78%) [28]. These characteristics enable ertugliflozin to be a potent SGLT2 inhibitor. This meta-analysis demonstrated 0.62% and 0.69% greater reductions in HbA1c from the baseline with ertugliflozin 5 mg and ertugliflozin 15 mg, respectively, than the placebo; there was no statistical difference in HbA1c lowering with ertugliflozin 5 mg and ertugliflozin 15 mg. In the meta-analyses of placebo-controlled trials, placebo-subtracted WMDs of HbA1c were −0.46% for dapagliflozin [29], −0.63% for canagliflozin 100 mg, −0.80% for canagliflozin 300 mg [30], −0.61% for empagliflozin 10 mg, and −0.63% for empagliflozin 25  mg [31]. Furthermore, McNeill et al., in their network meta-analysis, found that ertugliflozin 5 mg was superior to dapagliflozin 5 mg when added to metformin monotherapy. Moreover, ertugliflozin 15 mg had better glycemic efficacy than dapagliflozin 10 mg and empagliflozin 25 mg when added to diet/exercise and to metformin monotherapy [32]. Similar to us, higher doses of canagliflozin and ertugliflozin did not achieve higher HbA1c reductions in placebo-controlled trials [30, 31]. Ertugliflozin was as good as other SGLT2is in reducing FPG and the proportion of patients achieving HbA1c < 7% [29–32]. Higher doses of canagliflozin and ertugliflozin did not earn higher FPG reductions, and the percentage of people achieving HbA1c < 7% in placebo-controlled trials; similar dose-responsive differences were also absent for ertugliflozin in this meta-analysis [30, 31].

SGLT2is reduce body weight from 1 to 5 kg; this weight loss might be related to volume depletion and glucose excretion in the kidneys by SGLT2is, resulting in noticeable calorie loss [1]. In this meta-analysis, ertugliflozin was similarly effective in weight reduction to other SGLT2is [29–31]. Moreover, ertugliflozin 15 mg achieved 0.22 kg more weight reduction than ertugliflozin 5 mg. In placebo-controlled trials, canagliflozin 300  mg achieved greater weight reduction than canagliflozin 100  mg dosage group, whereas weight reductions were similar with empagliflozin 5 mg and 15 mg [30, 31]. BP reduction with SGLT2is results from natriuresis and osmotic diuresis initially and from local renin–angiotensin system inhibition later on [1]. In this meta-analysis, ertugliflozin showed significant SBP and DBP reductions, comparable to other SGLT2is [1, 29–31]. SGLT2is are considered to be either lipid-friendly or lipid-neutral drugs [1]. This meta-analysis found a nonsignificant, small increment in LDL-C and a significant increase in HDL-C with ertugliflozin compared to placebo. In a meta-analysis by Sánchez-García et al., canagliflozin, dapagliflozin, and empagliflozin significantly increased total cholesterol, LDL-cholesterol, non-HDL-cholesterol, and HDL-cholesterol and decreased triglyceride levels than placebo [33]. In another meta-analysis by Xiong et al., canagliflozin 100 and 300 mg showed similar increments in LDL-C (nonsignificant) and HDL-C (significant) [30]. Such an increment in LDL-C may result from natriuresis-induced hemoconcentration and decreased hepatocyte expression of LDL receptors. The increase in HDL-C and the decrease in triglyceride levels could be related to improving insulin sensitivity and secretion [33]. Moreover, the CV benefit of ertugliflozin has been well-reported by previous studies. Compared with gliptins, SGLT2is, including ertugliflozin, were associated with a significantly lower risk of new-onset syncope in patients with T2D, regardless of age, gender, glycemic control status, and comorbidity burden in a large-scale retrospective cohort study [34]. In a meta-analysis by Zhang et al., ertugliflozin and other SGLT2is did not decrease the risk of atrial fibrillation occurrence for patients with cardiometabolic diseases or risk factors [35].

The meta-analysis provides quite reassuring safety data for ertugliflozin, evidenced by no differences in any AEs, AEs related to the study drug, AEs leading to discontinuation of the study drug, serious AEs, serious AEs related to the study drug, and AEs leading to death between ertugliflozin and placebo groups. These findings are identical to the previous meta-analysis of ertugliflozin by Liu et al. [10]. In the meta-analysis by Xiong et al., canagliflozin showed similar results except higher AEs related to the study drug with canagliflozin and higher serious AE with placebo [30]. Like previous reports with ertugliflozin and other SGLT2is, we also found higher risks of GMI in both men and women [5, 9–11, 29–31]. Ertugliflozin and placebo showed comparable risks of UTI in this meta-analysis; previous meta-analyses reported similar risks [9–11]. Moreover, UTI risk was not increased with canagliflozin and empagliflozin [5, 30, 31], but dapagliflozin imparted higher UTI risk than placebo [5]. Hypovolemia risks were similar in the ertugliflozin and the placebo groups; previous meta-analyses with ertugliflozin and other SGLT2is reported similar risks [3, 9–11]. Except for the higher risk of documented hypoglycemia with ertugliflozin 15 mg, this meta-analysis found similar risks of symptomatic and documented hypoglycemia in the ertugliflozin and the placebo groups. Previous meta-analyses, including ertugliflozin and other SGLT2is, also showed no differences in symptomatic and severe hypoglycemic risks compared to placebo. Furthermore, in this meta-analysis, ertugliflozin 5 mg and ertugliflozin 15 mg imparted similar risks of the AEs as mentioned earlier. Similarly, the risks of GMI, UTI, hypovolemia, and hypoglycemia did not differ with empagliflozin 10 mg and empagliflozin 25 mg in placebo-controlled trials [31].

### 4.1. Strengths and Limitations

The main strength of this meta-analysis is the incorporation of a large population from a fairly good number of well-conducted clinical trials published to date; all were RCTs and double-blind trials. We have analyzed the subgroup differences between the two doses of ertugliflozin for the efficacy and safety outcomes and also changes in LDL-C and HDL-C and compared AEs in the ertugliflozin and placebo groups; such reports are missing in the previous meta-analyses. There are also several limitations. The study by Cannon et al. accounted for ~75% of the subjects in the meta-analysis, thus driving most outcomes [17]. High heterogeneity was observed for the HbA1c and FPG; the certainty of evidence generated for the primary outcome, HbA1c, was low.

## 5. Conclusions

This meta-analysis provides reassuring data on glycemic efficacy with good safety and tolerability of ertugliflozin over an extended period of clinical use in a diverse group of patients with T2D. Ertugliflozin increased GMI events, as observed with other SGLT2is in the previous studies. Ertugliflozin 5 mg and 15 mg have similar efficacy and safety profiles except for greater weight reduction with ertugliflozin 15 mg.

## Figures and Tables

**Table 1:** The basic characteristics of the included randomized controlled trials and participants.

**Trial registration no.**	**Study ID**	**Major baseline characteristics of the study subjects**	**Study arms**	**N**	**Age (years, ** **m** **e** **a** **n** ± SD**)**	**Female (%)**	**Duration of T2DM (years, mean (range) or ** **m** **e** **a** **n** ± SD)	**Baseline HbA1c (%)**	**Study duration**
NCT01059825	Amin et al. [16]	• Background AHA: Metformin • Those with abnormal ECG, CHF, and eGFR < 50 mL/min/1.73 m^2^ were excluded	Ertugliflozin 1 mg	54	53.1 ± 9.1	37.0	6.3 (0.1–24.0)	8.01 ± 0.17	12 weeks
			Ertugliflozin 5 mg	55	54.7 ± 7.7	25.5	6.7 (0.3–30.0)	7.88 ± 0.13	
			Ertugliflozin 10 mg	55	57.3 ± 6.5	43.6	6.1 (0.2–20.0)	8.13 ± 0.17	
			Ertugliflozin 25 mg	55	54.2 ± 8.8	32.7	6.0 (0.3–18.2)	8.30 ± 0.16	
			Sitagliptin	55	53.3 ± 10.7	27.3	6.3 (0.3–20.0)	8.24 ± 0.15	
			Placebo	54	54.0 ± 8.1	44.4	6.4 (0.3–20.5)	8.08 ± 0.14	

NCT01986881	Cannon et al. [17]	• Background AHA: Not mentioned (SOC treatment) • All had ASCVD	Ertugliflozin 5 mg	2746	64.4 ± 8.1	29.7	12.9 ± 8.3	8.2 ± 1.0	3.5 years^a^
			Ertugliflozin 15 mg	2747					
			Placebo	2745	64.4 ± 8.0	30.7	13.1 ± 8.4	8.2 ± 0.9	

NCT02036515	Dagogo-Jack et al. [18]	• Background AHA: Metformin and sitagliptin • Those with abnormal ASCVD, CHF, and eGFR < 60 mL/min/1.73 m^2^ were excluded	Ertugliflozin 5 mg	156	59.2 ± 9.3	48.1	9.9 ± 6.1	8.1 ± 0.9	Phase A: 26 weeks^b^ Phase B: 26 weeks
			Ertugliflozin 15 mg	153	59.7 ± 8.6	46.4	9.2 ± 5.3	8.0 ± 0.8	
			Placebo	153	58.3 ± 9.2	34.6	9.4 ± 5.6	8.0 ± 0.9	

NCT01986855	Grunberger et al. [19]	• Background AHA: Standard AHAs, including insulin and SUs • All had stage 3 CKD (eGFR ≥ 30 to < 60 mL/min/1.73 m^2^)	Ertugliflozin 5 mg	158	66.7 ± 8.3	46.8	14.9 ± 9.0	8.2 ± 1.0	Phase A: 26 weeks^b^ Phase B: 26 weeks
			Ertugliflozin 15 mg	155	67.5 ± 8.5	51.6	14.5 ± 8.5	8.2 ± 0.9	
			Placebo	154	67.5 ± 8.9	53.2	13.1 ± 8.1	8.1 ± 0.9	

NCT02630706	Ji et al. [20]	• Background AHA: Metformin • Those with eGFR < 55 mL/min/1.73 m^2^ were excluded	Ertugliflozin 5 mg	170	56.1 ± 9.0	44.1	7.0 ± 5.0	8.1 ± 0.9	26 weeks
			Ertugliflozin 15 mg	169	56.3 ± 9.3	42.0	7.5 ± 5.1	8.1 ± 0.9	
			Placebo	167	56.9 ± 9.0	47.3	6.4 ± 5.1	8.1 ± 1.0	

NCT02033889	Rosenstock et al. [21]	• Background AHA: Metformin • Those with eGFR < 55 mL/min/1.73 m^2^ were excluded	Ertugliflozin 5 mg	207	56.6 ± 8.1	53.1	7.9 ± 6.1	8.1 ± 0.9	Phase A: 26 weeks^b^ Phase B: 78 weeks
			Ertugliflozin 15 mg	205	56.9 ± 9.4	54.6	8.1 ± 5.5	8.1 ± 0.9	
			Placebo	209	56.5 ± 8.7	53.1	8.0 ± 6.3	8.2 ± 0.9	

NCT01958671	Terra et al. [22]	• Background AHA: Diet and exercise • Those with H/O CV event within 3 months, and eGFR < 55 mL/min/1.73 m^2^ were excluded	Ertugliflozin 5 mg	156	56.8 ± 11.4	42.9	5.11 ± 5.09	8.16 ± 0.88	Phase A: 26 weeks^b^ Phase B: 26 weeks
			Ertugliflozin 15 mg	152	56.2 ± 10.8	40.8	5.22 ± 5.55	8.35 ± 1.12	
			Placebo	153	56.1 ± 10.9	46.4	4.63 ± 4.52	8.11 ± 0.92	

Abbreviations: AHA = antihyperglycemic agents, ASCVD = atherosclerotic cardiovascular disease, CHF = congestive heart failure, CKD = chronic kidney disease, CV = cardiovascular, ECG = electrocardiogram, eGFR = estimated glomerular filtration rate, SOC = standard of care treatment, SD = standard deviation, SU = sulfonylurea.

^a^Changes in HbA1c, body weight, and systolic blood pressure were reported at Week 18.

^b^Placebo-controlled period, included in the meta-analysis.

**Table 2:** The basic characteristics of the excluded randomized controlled trials and participants.

**Study ID**	**Study duration**	**Reason of exclusion**	**Study arms**	**N**	**Age (years, ** **m** **e** **a** **n** ± **SD)**	**Baseline HbA1c (%)**	**Change from baseline in HBa1c (%)**
Aronson et al. [26]	52 weeks	• Extension study of Terra 2017 • After the 26-week, placebo-controlled period, metformin was added to the placebo group	Ertugliflozin 5 mg	156	56.8 ± 11.4	8.2 ± 0.9	−0.9 ± 0.1, mean ± SE −0.7 (−0.9, −0.6), LSM (96% CI)
			Ertugliflozin 15 mg	152	56.2 ± 10.8	8.4 ± 1.1	−1.0 ± 0.1, mean ± SE −0.9 (−1.0, −0.7), LSM (96% CI)
			Placebo + metformin	153	56.1 ± 10.9	8.1 ± 0.9	−1.0 ± 0.1, mean ± SE

Gallo et al. [27]	104 weeks	• Extension study of Rosenstock 2018 • After the 26-week, placebo-controlled period, Glimepiride Placebo or Glimepiride was added	Ertugliflozin 5 mg + glimepiride Placebo	207	56.6 ± 8.2	8.06 ± 0.89	−0.6 ± 0.1, mean ± SE −0.50 (−0.63, −0.37), LSM (96% CI)
			Ertugliflozin 15 mg + glimepiride placebo	205	56.9 ± 9.4	8.13 ± 0.93	−0.9 ± 0.1, mean ± SE −0.76 (−0.90, −0.63), LSM (96% CI)
			Placebo + glimepiride	209	56.5 ± 8.7	8.17 ± 0.90	−0.6 ± 0.1, mean ± SE

Hollander et al. [23]	104 weeks	• No placebo-control group	Ertugliflozin 5 mg + metformin ≥ 1500 mg	445	58.7 ± 9.8	7.8 ± 0.6	−0.3 (−0.4, −0.2), LSM (96% CI)
			Ertugliflozin 15 mg + metformin ≥ 1500 mg	435	58.0 ± 9.9	7.8 ± 0.6	−0.4 (−0.5, −0.3), LSM (96% CI)
			Glimepiride up to 6 or 8 mg + metformin ≥ 1.5 mg	435	57.9 ± 9.1	7.8 ± 0.6	−0.4 (−0.5, −0.3), LSM (96% CI)

Miller et al. [25]	26 weeks	• Ertugliflozin plus Sitagliptin was compared to placebo	Ertugliflozin 5 mg + sitagliptin 100 mg	98	56.4 ± 9.3	8.9 ± 0.9	−0.4 (−0.7, −0.2), LSM (96% CI)
			Ertugliflozin 15 mg + sitagliptin 100 mg	96	56.1 ± 10.1	9.0 ± 0.9	−1.6 (−1.8, −1.4), LSM (96% CI)
			Placebo	97	54.3 ± 10.3	9.0 ± 0.9	−1.7 (−1.9, −1.5), LSM (96% CI)

Pratley et al. [24]	52 weeks	• No placebo-control group	Ertugliflozin 5 mg	250	55.1 ± 10.1	8.6 ± 1.0	−1.0 (−1.1, −0.8), LSM (96% CI)
			Ertugliflozin 15 mg	248	55.3 ± 9.5	8.6 ± 1.0	−0.9 (−1.1, −0.8), LSM (96% CI)
			Sitagliptin 100 mg	247	54.8 ± 10.7	8.5 ± 1.0	−0.8 (−1.0, −0.7), LSM (96% CI)
			Ertugliflozin 5 mg + sitagliptin 100 mg	243	55.2 ± 10.4	8.6 ± 1.0	−1.4 (−1.5, −1.2), LSM (96% CI)
			Ertugliflozin 15 mg + sitagliptin 100 mg	244	55.1 ± 9.8	8.6 ± 1.0	−1.4 (−1.5, −1.3), LSM (96% CI)

Abbreviations: CI = confidence interval, LSM = least squares mean, SD = standard deviation, SE = standard error.

**Table 3:** Summary of findings.

**Outcomes**	**Anticipated absolute effects** **(95% CI)**		**Relative effect (95% CI)**	**No. of participants (studies)**	**Certainty of the evidence (GRADE)**
	**Risk with placebo**	**Risk with ertugliflozin**			
Change in HbA1c-ertugliflozin 5 mg	The mean change in HbA1c −0.18%	MD 0.62% lower (0.8 lower to 0.44 lower)	—	7283 (7 RCTs)	⨁⨁◯◯ Low^a^
Change in HbA1c-ertugliflozin 15 mg	The mean change in HbA1c −0.19%	MD 0.69% lower (0.91 lower to 0.47 lower)	—	7161 (6 RCTs)	⨁⨁◯◯ Low^a^
Any AEs-ertugliflozin 5 mg	779 per 1,000	777 per 1000 (756 to 796)	OR 0.99 (0.88 to 1.11)	7283 (7 RCTs)	⨁⨁⨁⨁ High
Any AEs-ertugliflozin 15 mg	782 per 1,000	770 per 1000 (744 to 794)	OR 0.93 (0.81 to 1.07)	7162 (6 RCTs)	⨁⨁⨁⨁ High
Serious AEs-ertugliflozin 5 mg	282 per 1,000	320 per 1000 (230 to 428)	OR 1.20 (0.76 to 1.91)	7283 (7 RCTs)	⨁⨁⨁⨁ High
Serious AEs-ertugliflozin 15 mg	286 per 1,000	271 per 1000 (233 to 313)	OR 0.93 (0.76 to 1.14)	7162 (6 RCTs)	⨁⨁⨁⨁ High
GMI (women)-ertugliflozin 5 mg	22 per 1,000	62 per 1000 (40 to 93)	OR 2.95 (1.88 to 4.62)	2439 (6 RCTs)	⨁⨁⨁⨁ High
GMI (women)-ertugliflozin 15 mg	22 per 1,000	78 per 1000 (52 to 116)	OR 3.79 (2.45 to 5.87)	2468 (6 RCTs)	⨁⨁⨁⨁ High
GMI (men)-ertugliflozin 5 mg	10 per 1,000	40 per 1000 (26 to 60)	OR 3.99 (2.57 to 6.19)	4735 (6 RCTs)	⨁⨁⨁⨁ High
GMI (men)-ertugliflozin 15 mg	10 per 1,000	45 per 1000 (30 to 69)	OR 4.59 (2.96 to 7.11)	4694 (6 RCTs)	⨁⨁⨁⨁ High
UTI–ertugliflozin 5 mg	86 per 1,000	94 per 1000 (71 to 122)	OR 1.10 (0.81 to 1.47)	7283 (7 RCTs)	⨁⨁⨁⨁ High
UTI–ertugliflozin 15 mg	87 per 1,000	88 per 1000 (53 to 141)	OR 1.01 (0.59 to 1.72)	7162 (6 RCTs)	⨁⨁⨁⨁ High
Symptomatic hypoglycemia-ertugliflozin 5 mg	228 per 1,000	226 per 1000 (206 to 245)	OR 0.99 (0.88 to 1.10)	7283 (7 RCTs)	⨁⨁⨁⨁ High
Symptomatic hypoglycemia-ertugliflozin 15 mg	231 per 1,000	242 per 1000 (172 to 328)	OR 1.06 (0.69 to 1.62)	7162 (6 RCTs)	⨁⨁⨁⨁ High

^a^High heterogeneity among the studies present.

## Data Availability

All data generated or analyzed during this study are available in this published article.
