# Supplementary material for: Efficacy and Safety of Ertugliflozin Compared to Placebo in Patients With Type 2 Diabetes: An Updated Systematic Review and Meta-Analysis
Source: J Diabetes Res. 2024 Sep 24;2024:5553327. doi: 10.1155/2024/5553327 (PMC11444800; doi:10.1155/2024/5553327)
Supplement: Supporting Information — Additional supporting information can be found online in the Supporting Information section. Table S1 Risk of bias assessment. [file 5553327.f1.doc]

**SUPPLEMENTARY MATERIALS**

**Supplementary Table S1.** Risk of bias assessment

| **Amin 2015** | **Risk of bias** | **Author Judgement** |
| --- | --- | --- |
|  |  |  |
| Random sequence generation (selection bias) | Low risk | Randomized, placebo- and active-controlled, parallel-group trial. Patients were randomized using a computer-generated random permuted block method. |
| Allocation concealment (selection bias) | Low risk | Random allocation using a computer-generated random permuted block method, double-dummy design. |
| Blinding of participants & personnel (performance bias) | Low risk | Double-blinded, double-dummy RCT. |
| Blinding of outcome assessment (detection bias) | Low risk | Double-blinded, double-dummy RCT. Some safety outcomes were assessed blindly. |
| Incomplete outcome data (attrition bias) | Low risk | Of the 328 subjects randomized, 12.8% discontinued the study. Missing outcome data balanced in numbers across intervention groups, with similar reasons for missing data across groups. |
| Selective reporting (reporting bias) | Low risk | All pre-specified outcomes are reported. |
| Other biases | High risk | Pfizer provided funding for this study.  All but one author are employees of Pfizer. |
| **Cannon 2020** | **Risk of bias** | **Author Judgement** |
| Random sequence generation (selection bias) | Low risk | Randomized, placebo-controlled trial. Randomization was performed at a central location with the use of an interactive voice-response system and was based on a computer-generated schedule with randomly permuted blocks, stratified according to geographic region. |
| Allocation concealment (selection bias) | Low risk | Random allocation using an interactive voice-response system based on a computer-generated schedule with randomly permuted blocks. |
| Blinding of participants & personnel (performance bias) | Low risk | Double-blind RCT. |
| Blinding of outcome assessment (detection bias) | Low risk | Double-blind RCT. A cardiovascular adjudication committee centrally adjudicated all the primary and secondary outcome events blindly. |
| Incomplete outcome data (attrition bias) | Low risk | ~87% completed the trial. Missing outcome data balanced in numbers across intervention groups, with similar reasons for missing data across groups. |
| Selective reporting (reporting bias) | Low risk | All pre-specified outcomes are reported. |
| Other biases | High risk | Supported by Merck Sharp & Dohme (a subsidiary of Merck) and Pfizer. Authors received financial benefits from many pharmaceutical companies, including the manufacturer of the drug studied. |
| **Dagogo-Jack 2017** | **Risk of bias** | **Author Judgement** |
| Random sequence generation (selection bias) | Low risk | Randomized, placebo-controlled, parallel-group trial. Randomization was done using a computer-generated randomization schedule. |
| Allocation concealment (selection bias) | Low risk | Random allocation using a computer-generated randomization schedule. |
| Blinding of participants & personnel (performance bias) | Low risk | Double-blind RCT. |
| Blinding of outcome assessment (detection bias) | Low risk | Double-blind RCT. Insufficient information about blinding of outcome assessment, but the review authors judge that the outcome measurement is not likely to be influenced by lack of blinding. |
| Incomplete outcome data (attrition bias) | Low risk | 462 of 464 patients randomized were analyzed. |
| Selective reporting (reporting bias) | Low risk | All pre-specified outcomes are reported. |
| Other biases | High risk | Funded by Merck & Co., Inc., Kenilworth, New Jersey. The sponsor was involved in the study design, collection, analysis and interpretation of data, as well as data checking of information provided in the manuscript. All but the first author are employees of the pharma companies. |
| **Grunberger 2018** | **Risk of bias** | **Author Judgement** |
| Random sequence generation (selection bias) | Low risk | Randomized, placebo-controlled, parallel-group trial. Randomization was implemented centrally using an interactive voice response system/integrated web response system. |
| Allocation concealment (selection bias) | Low risk | Eligible patients were randomized using a computer-generated schedule, with a block size of six. Ertugliflozin and placebo were packaged identically so that blinding was maintained. |
| Blinding of participants & personnel (performance bias) | Low risk | Double-blind RCT. |
| Blinding of outcome assessment (detection bias) | Low risk | Double-blind RCT. Laboratory assessments were performed at a central laboratory where analysts were blinded to treatment assignment. |
| Incomplete outcome data (attrition bias) | Low risk | 468 patients were randomized and 467 were treated; 417 (89.1%) completed phase A and 388 (82.9%) phase B. Missing outcome data balanced in numbers across intervention groups, with similar reasons for missing data across groups. |
| Selective reporting (reporting bias) | Low risk | All pre-specified outcomes are reported. |
| Other biases | High risk | Merck Sharp & Dohme Corp., a subsidiary of Merck & Co., Inc., Kenilworth, NJ, USA, in collaboration with Pfizer Inc, provided financial support for this study. All but the first author are employees of the pharma companies. |
| **Ji 2019** | **Risk of bias** | **Author Judgement** |
| Random sequence generation (selection bias) | Low risk | Randomized, placebo-controlled, parallel-group trial. Randomization was done using a computer-generated randomization code based on the method of random permuted blocks. |
| Allocation concealment (selection bias) | Low risk | The study subjects were assigned (1:1:1) to oral, once daily ertugliflozin 5 mg, 15 mg or placebo using a computer-generated randomization code based on the method of random permuted blocks. |
| Blinding of participants & personnel (performance bias) | Low risk | Double-blind RCT. |
| Blinding of outcome assessment (detection bias) | Low risk | Double-blind RCT. Laboratory assessments were performed at a central laboratory. Clinical adjudication committees, comprising external panels of independent physicians blinded to patient treatment assignments, evaluated cardiovascular events, fractures, pancreatitis and renal and hepatic events. |
| Incomplete outcome data (attrition bias) | Low risk | 92% patients completed the trial. Missing outcome data balanced in numbers across intervention groups, with similar reasons for missing data across groups. |
| Selective reporting (reporting bias) | Low risk | All pre-specified outcomes are reported. |
| Other biases | High risk | This study was sponsored by Merck Sharp & Dohme Corp., a subsidiary of Merck & Co., Inc., Kenilworth, NJ, USA, in collaboration with Pfizer Inc., New York, NY, USA. Most of the authors are employees of the pharma companies. |
| **Rosenstock 2018** | **Risk of bias** | **Author Judgement** |
| Random sequence generation (selection bias) | Low risk | Randomized, parallel-group trial. Randomization was done using a computer-generated randomization code based on the method of random permuted blocks. |
| Allocation concealment (selection bias) | Low risk | Participants were assigned (1:1:1) to placebo, ertugliflozin 5 mg or ertugliflozin 15 mg using a computer-generated randomization code based on the method of random permuted blocks. |
| Blinding of participants & personnel (performance bias) | Low risk | Double-blind RCT. |
| Blinding of outcome assessment (detection bias) | Low risk | Double-blind RCT. Insufficient information about blinding of outcome assessment, but the review authors judge that the outcome measurement is not likely to be influenced by lack of blinding. |
| Incomplete outcome data (attrition bias) | Low risk | The proportion of participants who discontinued study medication was 9.1%, 2.9% and 7.3% in the placebo, ertugliflozin 5-mg and 15-mg groups, respectively. Missing outcome data balanced in numbers across intervention groups, with similar reasons for missing data across groups. |
| Selective reporting (reporting bias) | Low risk | All pre-specified outcomes are reported. |
| Other biases | High risk | The study was funded by Pfizer, Inc., New York, New York USA and Merck & Co., Inc., Kenilworth, New Jersey USA. Five of the authors are employees of the pharma companies, others are financially benefited from the pharma companies. |
| **Terra 2017** | **Risk of bias** | **Author Judgement** |
| Random sequence generation (selection bias) | Low risk | Randomized, placebo-controlled, parallel-group trial. Randomization was done based on a computer-generated randomization code using the method of random permuted blocks. |
| Allocation concealment (selection bias) | Low risk | Patients were randomly assigned (1:1:1) via an interactive automated system to placebo, ertugliflozin 5 mg or ertugliflozin 15 mg, based on a computer-generated randomization code using the method of random permuted blocks. |
| Blinding of participants & personnel (performance bias) | Low risk | Double-blind RCT. |
| Blinding of outcome assessment (detection bias) | Low risk | Double-blind RCT. Insufficient information about blinding of outcome assessment, but the review authors judge that the outcome measurement is not likely to be influenced by lack of blinding. |
| Incomplete outcome data (attrition bias) | Low risk | Of the 4611 randomized patients, 459 completed the study on study medication. |
| Selective reporting (reporting bias) | Low risk | All pre-specified outcomes are reported. |
| Other biases | High risk | Funded by Merck Sharp & Dohme Corp., a subsidiary of Merck & Co., Inc.,  Kenilworth, NJ USA and Pfizer Inc. Seven of the authors are employees of the pharma companies, others are financially benefited from the pharma companies. |
